# Seat of the Soul!

**Published:** 1830-04-01

**Authors:** 


### Seat of the Soul.

We are afraid that, between "medical physics," and "medical metaphysics," *common physic*, by which so many of us "live, and move, and have our being," will fall to the ground! We all know the disputes that have existed respecting the seat of the soul, the mind, the intellectual principle, or whatever other name we chuse to apply to that *divinæ particula auræ* which is the boast of proud man. Most people, however, imagined that the brain was the seat of thought and the throne of intellect—probably from the vulgar fact that, whenever the brain was removed, by accident or design, all intellectual operations ceased—at least as far as MAN is concerned. It was reserved for the year 1830, to discover that a man, or, at all events, a woman, may live, and think, and shew the highest exertions of intellect after the seat and the source of the mind is removed. Dr. Pring, of Bath, in his recent work on the "intellectual and moral relations," has shewn a wonderful "alacrity at sinking," by precipitating the seat of the soul, and "origin of mind," from the pineal gland, or other portion of the encephalon, down to that infernal region, the PELVIS, and locating it (as brother Jonathan would say) in the ovarium!—Really the air or the waters of BATH seem to have a most powerful tendency to the BATHOS. But we must let the brilliant ovarian hypothesis be *delivered* (cross-birth or breech-presentation as it is) by the crotchets of the author.

"Supposing it to be conceded, that the mind, like the vital properties which subserve organic purposes, is derived from parents; in the case of the maternal ovum, to which our considerations are now chiefly confined, the following modes may be suggested by which the intellectual principle is possessed by this rudiment: either, first, the ovum is endowed with mind by a local function of the ovarium, in which it is produced; or, second, this seat (the ovarium) is a centre, to which the maternal properties tend, whether those of the intellectual, or of the organic system.

"Without discussing these alternatives at any great length, it may be observed, that if the formation of the ovum were the result merely of a local function of the ovarium, there is no reason why peculiarities of parents, possessed by distant seats, should be perpetuated in the offspring; and the participation of the offspring in such peculiarities, seems a sufficient proof, that the endowments of the ovum are not merely from properties of the ovarium, but that they are communicated from all parts of the parent; and that the ovum is a miniature representation of the properties both of the organic, animal, and intellectual systems, which occupy respectively the structures of the parent."

There is one difficulty attendant on this hypothesis—but what hypothesis is free from difficulties?—and that is this. As our original progenitor, ADAM, had neither ovarium nor ovum—and as these bodies have never since been discovered, except on very rare occasions, in the bodies of men, it is very difficult to account for the features, the propensities, and the moral or physical qualities of the father in the child. If the ovum be a "miniature representation" of the properties, propensities, &c. of the parent, it follows that we are all the veritable descendents of mother EVE *alone*, and consequently inheritors of *her* propensities—Adam having no ovaria. Upon mature consideration, we are inclined very much to favour Dr. Pring's hypothesis. EVE was evidently the origin of SIN—for poor ADAM was merely a second-hand performer in the drama of the Serpent and the Apple: and as SIN has characterized the whole of the hopeful progeny, from Mother Eve down to Mother ——— in Chandos-street, Dr. Pring has now furnished us with a KEY to the whole mystery!

